# Childhood adversity and peer influence in adolescent bullying perpetration

**DOI:** 10.1038/s41598-024-81978-8

**Published:** 2024-12-28

**Authors:** Lawrence E. Ugwu, Kedibone J. Ramadie, Wojujutari Kenni Ajele, Erhabor Sunday Idemudia

**Affiliations:** 1 Faculty of Humanities, North-West University Mafikeng, Mafikeng, South Africa; 2 School of Psycho-Social Education, Subject Group Educational Psychology, Faculty of Education, North-West University, Mahikeng, South Africa

**Keywords:** Bullying, Childhood adversity, Peer influence, Personality traits, South African adolescents, Social learning theory, Psychology, Human behaviour

## Abstract

Bullying among South African adolescents is a critical public health issue. This study explores the relationship between childhood adversity, peer influence, and personality traits in predicting bullying perpetration. Data from 769 high school learners were analysed using Structural Equation Modelling. Findings indicate that childhood adversity predicts bullying perpetration, mediated by peer influence and moderated by personality traits like conscientiousness, extraversion, and emotional stability. These results support Social Learning Theory, emphasising observed behaviours and peer dynamics in bullying. Practical implications include targeted interventions addressing childhood adversity and fostering positive peer interactions and personality development.

## Introduction

Bullying perpetration among adolescents in South Africa represents a significant public health issue with profound social and psychological implications^1^. Bullying perpetration, aggressive behaviour intended to harm or disturb another individual^2^, is influenced by the unique socio-cultural dynamics and historical context of South Africa, which affect male and female adolescents differently^3^. Engaging in bullying behaviours has been associated with various negative outcomes for perpetrators, including an increased risk of future aggression, delinquency, and antisocial behaviour^4,5^. While much of the literature focuses on the victims of bullying, understanding the factors contributing to bullying perpetration is crucial for developing effective interventions.

Research indicates that bullying perpetrators may experience social development impairments^6^ and declines in academic performance^7^. Some studies suggest that perpetrators may also suffer from mental health issues such as depression and anxiety, although findings are mixed and often context-dependent^8,9^. The long-term consequences for perpetrators can extend into adulthood, affecting social relationships and increasing the risk of criminal behaviour^10,11^.

The relationship between bullying perpetration and childhood adversity is well-established. Adverse experiences such as corporal punishment, sexual abuse, and hostile home environments have been found to significantly predict aggressive behaviours, including bullying perpetration^12–16^. For instance, Hunter and Morrell^17^ found a high prevalence of corporal punishment in South African schools correlating with increased student aggression, with notable gender differences. Children exposed to hostile home environments may develop impaired emotional regulation and social skills, raising the likelihood of engaging in bullying behaviours^18^. Emotional and behavioural dysregulation resulting from childhood adversity often manifests as aggression in school settings, contributing to a cycle of violence^19,20^.

Peer influence plays a critical role in how early adverse experiences translate into bullying perpetration. Adolescents with histories of adversity may be more susceptible to negative peer influences, which can reinforce aggressive behaviours^21^. Peer groups can provide a social context where bullying behaviours are modelled, reinforced, or even rewarded, thereby increasing the likelihood of an individual engaging in such behaviours^22^. However, the role of personality traits in these dynamics remains underexplored, especially among South African adolescents.

Personality traits—including openness to experience, conscientiousness, extraversion, agreeableness, and emotional stability—may significantly affect how individuals respond to adversity and peer influences^23^. For example, higher agreeableness and emotional stability have been associated with lower levels of aggression and bullying perpetration^24^. Individuals high in these traits may exhibit greater resilience, potentially reducing their likelihood of engaging in bullying behaviours despite adverse experiences and negative peer influences^25^. Conversely, high extraversion or low conscientiousness personality may exacerbate the risk of bullying perpetration^26^.

Despite extensive research on bullying in South Africa, there remains a gap in understanding how childhood adversity, peer influence, and personality traits interact to influence bullying perpetration. This gap is particularly pronounced in sub-Saharan Africa, where diverse socio-cultural dynamics profoundly impact adolescent behaviour. Understanding how individual personality traits may mitigate or exacerbate bullying perpetration stemming from early adversity is crucial for developing effective interventions.

This study aims to explore the relationship between childhood adversity and bullying perpetration, focusing on peer influence as a mediator and personality traits as moderators among South African adolescents across genders. The central research question is: How do personality traits (openness to experience, conscientiousness, extraversion, agreeableness, and emotional stability) moderate the relationship between childhood adversity (punishment, sexual abuse, and negative home atmosphere) and bullying perpetration, as mediated by peer influence, across genders in the South African context?

Exploring these interactions is vital for developing culturally relevant and effective interventions in South Africa. Insights into how peer influence and personality traits interact within the framework of childhood adversity can guide policymakers and educators in crafting targeted bullying prevention and intervention strategies that address the specific needs of bullying perpetrators^27^.

## Literature review

### Definition of bullying

Bullying is defined as intentional, repetitive, aggressive behaviour that involves an imbalance of power between the perpetrator and the victim^2^. It encompasses physical aggression, verbal harassment, relational aggression, and cyberbullying. Bullying has significant adverse effects on both victims and perpetrators, including mental health issues, academic difficulties, and social problems^28^. Understanding the factors contributing to bullying perpetration is essential for developing effective prevention and intervention strategies.

### Childhood adversity and bullying perpetration

Childhood adversity, such as physical abuse, emotional abuse, neglect, and exposure to domestic violence, has been consistently linked to an increased likelihood of bullying perpetration in adolescence^29^. Studies have shown that these adverse experiences can lead to a higher propensity for bullying perpetration across both genders. For instance, a study by Putnam et al.^30^ found that sexual abuse, physical abuse, and neglect were significantly correlated with aggressive behaviours in both boys and girls. Similarly, research by Jewkes et al.^31^ demonstrated that various forms of childhood adversity, such as emotional and physical abuse, were linked to increased behavioural problems, including bullying, in both male and female adolescents^30,31^.

In a meta-analysis, Asscher et al.^32^ explored the relationship between childhood abuse and subsequent antisocial behaviour, including bullying, finding significant associations across genders. These findings are supported by Sideli et al.^33^, who highlighted that adverse childhood experiences are robust predictors of bullying behaviours in both boys and girls^32^.

The reviewed paper by Li et al.^34^ supports the link between personality traits and bullying, indicating that personality profiles such as ‘Undercontrolled’ (characterised by low Conscientiousness and high Neuroticism and Extraversion) are more likely to engage in bullying behaviours^34^.

While these studies provide a broad understanding, limited research focuses explicitly on Sub-Saharan Africa, especially among South African adolescents. This study aims to fill this gap by examining how childhood adversity influences bullying perpetration in this specific demographic, considering the unique socio-cultural context of South Africa.

Theoretical frameworks provide insights into why this association exists. Social Learning Theory^35^ posits that children learn behaviours through observing and imitating others, particularly significant figures like parents or caregivers. Children exposed to aggressive behaviour at home may internalise these actions as acceptable ways to interact with others. For instance, if a child witnesses or experiences violence, they may adopt similar behaviours when interacting with peers.

Attachment Theory^36^ suggests that early adverse experiences can disrupt the development of secure attachments, leading to difficulties in emotional regulation and social relationships. Children with insecure attachments may struggle with empathy and exhibit externalising behaviours, including bullying, as a means of expressing unmet emotional needs.

Empirical studies support these theoretical perspectives. Shields and Cicchetti^37^ found that maltreated children were more likely to engage in bullying behaviours compared to non-maltreated peers. Similarly, Schwartz et al.^38^ reported that children who experienced physical abuse exhibited higher levels of aggression and bullying. These findings indicate that childhood adversity contributes to the development of maladaptive behaviours that manifest as bullying.

#### Hypothesis 1

Higher levels of childhood adversity (punishment, sexual abuse, and negative home atmosphere) are positively associated with bullying perpetration.

### Childhood adversity and susceptibility to peer influence

Adolescents who have experienced childhood adversity may be more susceptible to peer influence due to unmet emotional needs and impaired social skills. Social Control Theory^39^ suggests that weak bonds to conventional social institutions (like family and school) increase the likelihood of deviant behaviour influenced by peers. Adolescents lacking strong family support may seek acceptance and belonging in peer groups, making them more vulnerable to peer pressure.

Additionally, adverse experiences can hinder the development of self-regulation and increase the need for social approval^40^. Such adolescents might associate with deviant peers to fulfil emotional and social needs, inadvertently increasing their exposure to behaviours like bullying.

Empirical evidence highlights this association. Kim and Cicchetti^41^ found that maltreated children were more likely to affiliate with peers who engage in antisocial behaviour. Lansford et al.^42^ demonstrated that childhood abuse predicted higher susceptibility to peer influence in adolescence, leading to increased externalising behaviours.

#### Hypothesis 2

Higher levels of childhood adversity are positively associated with susceptibility to peer influence.

### Peer influence and bullying perpetration

Peer influence is a well-established predictor of bullying behaviour. Adolescents often model the behaviours of their peers, and negative peer influence can lead to increased instances of bullying. Research by Gwadz et al.^43^ and Sideli et al.^33^ supports this, showing that adolescents whom their peers heavily influence are more likely to engage in bullying. This relationship holds for both boys and girls. Jewkes et al.^31^ also noted that peer dynamics play a crucial role in the manifestation of bullying behaviour among adolescents. Those who experience negative peer influence are more likely to participate in bullying, regardless of gender^31,43^.

Research on peer influence and bullying has been less explored in the context of South African adolescents. This study aims to investigate this dynamic, considering the distinct social structures and peer relationships within South African schools.

Susceptibility to peer influence is a critical predictor of bullying perpetration. Adolescents often conform to peer norms to gain acceptance or avoid rejection^44^. Social Identity Theory^45^ explains that individuals derive part of their identity from group membership, and aligning with group norms—including aggressive behaviours like bullying—can enhance their social standing within the group.

Peer influence predicts bullying perpetration, particularly in environments where aggression is rewarded or considered a means to achieve social dominance^46^. Adolescents who are highly susceptible to peer influence may engage in bullying to fit in or elevate their status among peers.

Research supports this link. Espelage, Holt, and Henkel^47^ found that association with aggressive peers significantly predicted bullying behaviours. Their study emphasised that peer groups reinforce bullying by establishing norms that endorse such behaviour.

#### Hypothesis 3

Higher susceptibility to peer influence is positively associated with bullying perpetration.

### Peer influence as a Mediator

Children who experience high levels of adversity often seek acceptance and validation from peers, which makes them more susceptible to peer influence. This peer influence can lead to both positive and negative behaviours. Gwadz et al.^43^ found that children with histories of abuse and neglect were more likely to conform to peer behaviours, including risky and antisocial activities. This finding was consistent across genders. Wolff et al.^48^ further demonstrated that childhood adversities, such as physical and emotional abuse, were significantly associated with increased susceptibility to peer influence. This relationship was observed in male and female participants, indicating that childhood adversity leads to a heightened sensitivity to peer dynamics, irrespective of gender^43,48^.

Existing studies predominantly focus on Western contexts. This research will explore how childhood adversity affects peer influence among South African adolescents, providing insights into cultural and regional variations.

Rytioja^49^ found that peer delinquency influenced the relationship between childhood maltreatment and adolescent aggression. Hong and Espelage^50^ reported that peer norms and affiliations mediated the link between family abuse and bullying perpetration, highlighting the critical role of peer influence in this pathway.

The mediating role of peer influence in the relationship between childhood adversity and bullying perpetration can be understood through the Deviant Peer Contagion Theory^51^. This theory posits that adolescents who have experienced adversity may seek out peers with similar experiences or behaviours, increasing their exposure to and reinforcement of deviant behaviours like bullying.

The Social Development Model^52^ further explains that weak bonds with prosocial individuals and institutions due to childhood adversity can lead adolescents to affiliate with deviant peers, reinforcing aggressive behaviours.

#### Hypothesis 4

Peer influence mediates the relationship between childhood adversity and bullying perpetration.

### Personality traits as moderators

Personality traits within the Big Five framework can moderate the relationship between peer influence and bullying perpetration. Conscientiousness and agreeableness are associated with self-discipline, empathy, and cooperative behaviour, potentially buffering against negative peer influence^53^. Conversely, low levels of these traits may increase vulnerability to peer pressure and aggressive behaviours.

Extraversion may enhance exposure to social interactions and peer influence, possibly increasing the risk of engaging in bullying if aggressive behaviours are prevalent in the peer group. Emotional stability (the opposite of neuroticism) might affect how adolescents respond to peer pressure, with lower emotional stability potentially leading to higher susceptibility.

The Differential Susceptibility Hypothesis^53^ suggests that individuals differ in their sensitivity to environmental influences based on their personality traits. Adolescents with certain traits may be more or less affected by peer influence regarding bullying perpetration.

Empirical research supports these moderating effects. Jaruseviciute^54^ found that high conscientiousness and agreeableness moderated the association between peer influence and bullying, serving as protective factors. Kokkinos^55^ reported that personality traits influenced adolescents’ responses to peer pressure in the context of bullying.

#### Hypothesis 5

Personality traits (conscientiousness, extraversion, agreeableness, openness to experience, and emotional stability) moderate the relationship between peer influence and bullying perpetration.

### Mediated moderation: the interplay of peer influence and personality traits

Integrating the mediating role of peer influence and the moderating effects of personality traits provides a broader understanding of bullying perpetration. Mediated moderation occurs when the indirect effect of an independent variable on an outcome through a mediator varies across levels of a moderator^56^.

For example, an adolescent with a history of childhood adversity and low conscientiousness may be more susceptible to peer influence, increasing the likelihood of bullying. In contrast, high conscientiousness may buffer against peer influence, reducing the risk of bullying despite adverse experiences.

This framework aligns with ecological and developmental theories, which emphasise the interaction between individual characteristics and environmental factors^57^.

#### Hypothesis 6

The mediated relationship between childhood adversity and bullying perpetration through peer influence is moderated by personality traits.

## Current study

**Fig. 1:**
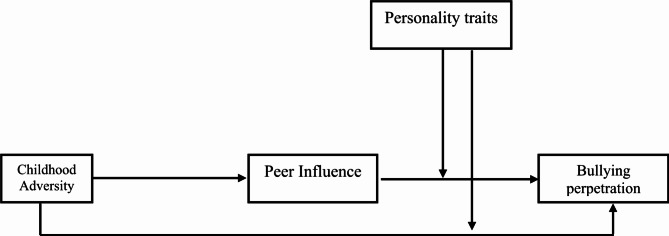
Conceptual framework of childhood adversity and bullying perpetration: the mediated moderating role of peer influence and personality traits (openness to experience, conscientiousness, extraversion, agreeableness and emotional stability).

The present study (see Fig. 1) aims to examine the complex interplay between childhood adversity, peer influence, and personality traits in predicting bullying perpetration among South African adolescents. Specifically, the research seeks to:

i. Investigate whether childhood adversity is associated with increased bullying perpetration.
ii. Examine the relationship between childhood adversity and susceptibility to peer influence.
iii. Assess whether susceptibility to peer influence predicts bullying perpetration.
iv. Determine if peer influence mediates the relationship between childhood adversity and bullying perpetration.
v. Explore whether personality traits moderate the relationship between peer influence and bullying perpetration.
vi. Evaluate whether personality traits moderate the indirect effect of childhood adversity on bullying perpetration through peer influence.

This study addresses these research questions, filling a significant knowledge gap in the South African context. There is a paucity of research examining the combined effects of childhood adversity, peer influence, and personality traits on bullying perpetration among South African adolescents. Understanding these relationships is crucial for developing culturally relevant interventions to reduce bullying and promote adolescent well-being in South Africa.

## Methods

### Participants

Participants were selected from the population of high school learners in secondary schools within the North-West Province of South Africa. The study focused on Grade 11 and Grade 12 learners to ensure sufficient reading comprehension for understanding the questionnaire items. A total of 769 learners participated in the study, comprising 356 (46.3%) females and 413 (53.7%) males.

A convenience sampling strategy was employed to select participants from the available schools. This non-probability sampling method was chosen due to logistical considerations and the accessibility of participants willing to partake in the study.

### Ethical considerations

Ethical approval was obtained from the Health Research Ethics Committee (HREC) of the Faculty of Health Sciences at North-West University (NWU), South Africa. Permissions were also secured from the Department of Education and the principals of the participating secondary schools. Informed consent was obtained from the parents or legal guardians of all participants. The study adhered to ethical guidelines outlined in the Declaration of Helsinki, ensuring participant confidentiality and anonymity.

### Instruments

#### Child abuse and trauma scale (CATS)

The Child Abuse and Trauma Scale (CATS), developed by Sanders and Becker-Lausen in 1995, was employed in this study to evaluate experiences of childhood adversity. This 38-item self-report questionnaire is designed to capture a range of adverse childhood experiences across three distinct subscales: Sexual Abuse, Punishment, and Neglect/Negative Home Atmosphere. The Sexual Abuse subscale assesses instances of sexual abuse occurring during childhood, while the Punishment subscale evaluates the frequency and severity of physical punishment, reflecting physical abuse experiences. The Neglect/Negative Home Atmosphere subscale measures emotional neglect and the overall negativity of the home environment.

Participants respond to each item on a 5-point Likert scale, where responses range from 0 (“Never”) to 4 (“Always”), with higher scores indicating greater exposure to adverse experiences. CATS has demonstrated robust reliability and validity across various international contexts^58^ and has shown acceptable psychometric properties in South African research^59^, making it an effective tool for assessing childhood adversity in this setting.

#### Peer pressure scale

Peer influence was assessed using the 10-item Peer Pressure Scale developed by Clasen and Brown^60^. This scale measures susceptibility to peer pressure in areas such as peer activities, misconduct, and conformity to norms. Participants rate items on a 5-point Likert scale from 1 (Strongly Disagree) to 5 (Strongly Agree). Higher scores indicate higher level of peer influence. The scale has shown good reliability and validity in adolescent populations^61^. It has been adapted in South African studies with satisfactory psychometric properties^62^.

#### Illinois bully scale (IBS)

Bullying perpetration was measured using the Bullying subscale of the Illinois Bully Scale^63^. The IBS is an 18-item instrument, but only the 9-item Bullying subscale was utilised in this study. Participants indicate how often they engaged in specific bullying behaviours in the past 30 days on a 5-point Likert scale ranging from 0 (Never) to 4 (Seven or more times). Sample items include: “I upset other students for the fun of it” and “I spread rumors about other students.” The IBS has demonstrated reliability and validity^63^. In South Africa, the scale has been used with acceptable psychometric properties^64^, showing reliability coefficients above 0.80.

#### Ten-item personality inventory (TIPI)

Personality traits were assessed using the Ten-Item Personality Inventory (TIPI) by Gosling et al.^65^. The TIPI measures the Big Five personality dimensions (Openness to Experience, Conscientiousness, Extraversion, Agreeableness, and Emotional Stability). Each dimension is represented by two items (one positively worded and one negatively worded), rated on a 7-point Likert scale from 1 (Disagree Strongly) to 7 (Agree Strongly). Despite its brevity, the TIPI has demonstrated acceptable levels of reliability and validity^65^. South African studies have reported satisfactory psychometric properties when using the TIPI^66^.

#### Data collection procedures

Data were collected through an online survey platform, which facilitated ease of access and response flexibility. The school authorities distributed the survey link to participants through official communication channels like email and the school portal. Providing access over three weeks allowed participants to complete the survey at their convenience, which can enhance response rates and ensure a representative sample of participants^67^.

The survey included an informed consent form, demographic questions, and the primary study instruments. The informed consent form detailed the study’s purpose, the voluntary nature of participation, and confidentiality assurances, which align with ethical guidelines for research involving human participants^68^. After reviewing this information, participants provided electronic assent before the survey.

The online format also allowed for anonymity and confidentiality by omitting any collection of personally identifiable information. Studies show that online data collection is particularly effective in maintaining anonymity and enhancing the honesty and reliability of responses, especially in educational research^69^. The collected data were securely stored on an encrypted platform to protect participants’ privacy.

The study adopted a cross-sectional design, capturing participant data simultaneously. Cross-sectional surveys efficiently provide a “snapshot” of participants’ attitudes and behaviours, which can be valuable for studies focused on descriptive or exploratory aims^70^. Additionally, the three-week period for data collection reduced common method bias, allowing participants to respond at their own pace, reducing response pressure and potential consistency biases^71^.

### Data analysis

In this study, data analysis was conducted using SmartPLS 4.1.0.8, ideal for Structural Equation Modelling (SEM) with a Partial Least Squares (PLS) approach, suitable for complex models. The analysis entailed assessing the measurement model for internal consistency (Cronbach’s alpha and Composite Reliability), convergent validity (Average Variance Extracted), and structural model assessment through path coefficient estimation using bootstrapping. Model fit was evaluated using Chi-square, Confirmatory Fit Index (CFI), Standardized Root Mean Square Residual (SRMR) and Root Mean Square Error of Approximation (RMSEA), with thresholds indicating a good fit. Additionally, the mediation effects of peer influence on the relationship between childhood adversity and bullying perpetration and moderation effects of openness to experience were analysed, including a mediated moderation analysis to explore the conditional indirect effects at different openness levels.

## Results

### Data screening and preparation

The dataset comprising 769 participants was examined for accuracy and completeness. Missing data were minimal, with less than 1% missing responses per item. Little’s Missing Completely at Random (MCAR) test indicated that the data were missing completely at random (χ²(7) = 16.50, *p* = .42). Consequently, the Expectation-Maximisation (EM) algorithm was employed to estimate and impute the missing values, preserving the overall distribution and integrity of the dataset. Outlier detection was conducted using standardised z-scores for univariate outliers and Mahalanobis distance for multivariate outliers at an alpha level of 0.001. No significant outliers were identified, confirming that the data were suitable for subsequent analyses.

The final sample consisted of 769 high school learners from the North-West Province of South Africa. The mean age of participants was 15.86 years (SD = 1.92). Given the high correlation between age and grade, only age was emphasised in the analyses to avoid redundancy. The sample included 356 females (46.3%) and 413 males (53.7%) (see Table 1).

**Table 1:** Demographic characteristics of participants.

Characteristic	Category	Frequency	Percentage (%)
Gender	Female	356	46.3
	Male	413	53.7

The internal consistency of the instruments was assessed using Cronbach’s alpha coefficients. The subscales of the Child Abuse and Trauma Scale (CATS) demonstrated acceptable reliability: Punishment (α = 0.79), Sexual Abuse (α = 0.70), and Negative Home Atmosphere (α = 0.78). The Peer Pressure Scale exhibited good reliability with a Cronbach’s alpha of 0.75, indicating its effectiveness in assessing susceptibility to peer influence. The Bullying subscale of the Illinois Bully Scale showed strong internal consistency (α = 0.80), suggesting it effectively measures bullying perpetration.

The alpha coefficients for the Big Five personality dimensions of the Ten-Item Personality Inventory (TIPI) ranged from 0.72 to 0.76, indicating that the TIPI reliably measures extraversion, agreeableness, conscientiousness, emotional stability, and openness to experience.

Confirmatory Factor Analysis (CFA) was conducted on the CATS and TIPI to assess their construct validity. For the CATS, the CFA supported the three-factor structure of Punishment, Sexual Abuse, and Negative Home Atmosphere. The fit indices indicated an acceptable model fit: χ^²^(132) = 287.45, *p* < .001; Comparative Fit Index (CFI) = 0.95; Root Mean Square Error of Approximation (RMSEA) = 0.05; and Standardised Root Mean Square Residual (SRMR) = 0.04. These results confirm that the subscales adequately represent distinct but related aspects of childhood adversity.

Similarly, the TIPI’s five-factor structure of personality traits was confirmed through CFA. The model demonstrated acceptable fit indices: χ²(80) = 162.30, *p* < .001; CFI = 0.96; RMSEA = 0.04; and SRMR = 0.03. These findings validate the TIPI’s ability to capture the five major dimensions of personality within the sample.

**Table 2:** Means, standard deviations, and correlations among variables (part 1).

No.	Variable	M	SD	1	2	3	4	5
1	Age	15.86	1.92	–				
2	Punishment	11.33	3.45	0.13**	–			
3	Sexual Abuse	16.21	10.09	0.15**	0.30**	–		
4	Negative Home Atmosphere	4.26	4.77	0.08*	0.20**	0.58**	–	
5	Peer Influence	23.97	8.55	0.06	0.21**	0.37**	0.41**	–

*p* < .05 (*), *p* < .01 (**).

**Table 3:** Means, standard deviations, and correlations among variables (part 2).

No.	Variable	M	SD	6	7	8	9	10
6	Bullying perpetration	17.22	13.01	--				
7	Extraversion	8.03	2.99	0.06*	–			
8	Agreeableness	8.64	2.59	− 0.00	0.12**	–		
9	Conscientiousness	8.07	2.62	− 0.07*	0.16**	0.18**	–	
10	Emotional stability	8.30	3.02	− 0.08*	0.19**	0.14**	0.10**	–

*p* < .05 (*), *p* < .01 (**).

Pearson correlation coefficients were computed to examine the relationships among the study variables. The correlation matrix is presented in tables (see Tables 2 and 3). The analysis revealed significant positive correlations between the childhood adversity variables and bullying perpetration, supporting Hypothesis 1. Specifically, Punishment, Sexual Abuse, and Negative Home Atmosphere were all positively correlated with Bullying Perpetration (coefficients ranging from 0.15** to 0.39**, *p* < .01). Peer Influence was also positively correlated with Bullying Perpetration (*r* = .46**, *p* < .01), aligning with Hypothesis 3.

Personality traits demonstrated significant associations with bullying perpetration. Conscientiousness and Emotional Stability were negatively correlated with Bullying penetration, indicating that higher levels of these traits are associated with lower levels of bullying perpetration. Extraversion was positively correlated with Bullying penetration, suggesting that more extroverted adolescents are more likely to engage in bullying.

A structural equation model (SEM) was tested to examine the hypothesised relationships among childhood adversity, peer influence, personality traits, and bullying perpetration. The model included paths from the childhood adversity variables (Punishment, Sexual Abuse, Negative Home Atmosphere) to Peer Influence, Peer Influence, Bullying Perpetration, and direct paths from Personality Traits to Bullying Perpetration. Interaction terms between Peer Influence and Personality Traits were included to assess moderation effects. The model fit indices indicated a good fit to the data: χ²(450) = 785.60, *p* < .001; Comparative Fit Index (CFI) = 0.96; Root Mean Square Error of Approximation (RMSEA) = 0.03; and Standardised Root Mean Square Residual (SRMR) = 0.04. These indices suggest that the hypothesised model adequately represents the data.

**Table 4:** Direct, mediation and moderation coefficients.

	Complete	
Direct effects	Coeff	T stat
Agreeableness → BP	0.05	2.09*
Conscientiousness → BP	− 0.04	1.96*
Extraversion → BP	0.10	4.25**
NHA→ BP	0.13	8.74**
NHA→ PI	0.29	10.02**
ES→ BP	− 0.08	3.30**
Openness → BP	− 0.10	4.42**
PI→ BP	0.46	18.25**
CA-P→ BP	0.04	3.80**
CA-P→ PI	0.10	3.98**
CA-SA→ BP	0.08	4.90**
CA-SA→ PI	0.18	5.18**
Openness x PI→ BP	− 0.05	1.55
Extraversion x PI→ BP	0.12	3.75**
Conscientiousness x PI→ BP	0.05	2.03*
ESx PI→ BP	− 0.03	1.13
Agreeableness x PI→ BP	0.01	0.36
Conditional indirect effect		
CA-NHA → PI → BP conditional on openness (high)	0.01	0.76
CA-P → PI → BP conditional on openness (high)	0.01	0.09
CA-SA → PI → BP conditional on openness (high)	− 0.01	0.79
CA-NHA → PI → BP conditional on openness (low)	− 0.01	0.27
CA-P → PI → BP conditional on openness (low)	0.01	0.03
CA-SA → PI → BP conditional on openness (low)	0.01	0.27
CA-NHA → PI → BP conditional on conscientiousness (high)	− 0.04	2.90**
CA-P → PI → BP conditional on conscientiousness (high)	− 0.01	0.12
CA-SA → PI → BP conditional on conscientiousness (high)	0.03	2.75**
CA-NHA → PI → BP conditional on conscientiousness (low)	0.06	2.61**
CA-P → PI → BP conditional on conscientiousness (low)	0.01	0.12
CA-SA → PI → BP conditional on conscientiousness (low)	− 0.04	3.11**
CA-NHA → PI → BP conditional on extraversion (high)	− 0.02	0.86
CA-P → PI → BP conditional on Extraversion (high)	0.01	0.08
CA-SA → PI → BP conditional on extraversion (high)	0.01	0.85
CA-NHA → PI → BP conditional on extraversion (low)	0.02	1.30
CA-P → PI → BP conditional on extraversion (low)	0.01	0.10
CA-SA → PI → BP conditional on extraversion (low)	− 0.02	1.42
CA-NHA → PI → BP conditional on agreeableness (high)	0.01	0.39
CA-P → PI → BP conditional on agreeableness (high)	0.01	0.05
CA-SA → PI → BP conditional on agreeableness (high)	− 0.01	0.41
CA-NHA → PI → BP conditional on agreeableness (low)	0.01	0.34
CA-P → PI → BP conditional on agreeableness (low)	0.01	0.04
CA-SA → PI → BP conditional on agreeableness (low)	− 0.01	0.35
CA-NHA → PI → BP conditional on ES (high)	− 0.01	0.73
CA-P → PI → BP conditional on ES (high)	0.01	0.07
CA-SA → PI → BP conditional on ES (high)	0.01	0.72
CA-NHA → PI → BP conditional on ES (low)	0.02	0.95
CA-P → PI → BP conditional on ES (low)	0.01	0.09
CA-SA → PI → BP conditional on ES (low)	− 0.01	1.02

*p* < .05 (*), *p* < .01 (**); BP = Bully perpetration; CA-NHA = Childhood adversity- Neglect/Negative Home Atmosphere; CA-P = Childhood Adversity-Punishment; CA-SA = Childhood Adversity-Sexual abuse; PI = Peer influence.

The table above (see Table 4) showed significant direct effects between the childhood adversity variables and Peer Influence and Bullying penetration. Punishment (β = 0.10, *t* = 3.98, *p* < .001), Sexual Abuse (β = 0.18, *t* = 5.18, *p* < .001), and Negative Home Atmosphere (β = 0.29, *t* = 10.02, *p* < .001) positively predicted Peer Influence. The childhood adversity indicates a medium effect size (*f*^2^ = 0.18). In turn, Peer Influence had a statistically significant positive effect on Bullying Penetration (β = 0.46, *t* = 18.25, *p* < .001). The peer influence indicates a small effect size (*f*^2^ = 0.03).

Regarding the direct effects of personality traits on Bullying penetration, Extraversion had a significant positive effect (β = 0.10, *t* = 4.25, *p* < .01), indicating that higher levels of extraversion are associated with increased bullying perpetration. Conscientiousness negatively predicted Bullying Perpetration (β = -0.04, *t* = -1.96, *p* < .05), suggesting that higher conscientiousness is associated with reduced bullying perpetration. Openness to Experience (β = -0.10, *t* = -4.42, *p* < .01) and Emotional Stability (β = -0.08, *t* = -3.30, *p* = .001) also had significant negative effects on Bullying Perpetration. The personality traits indicate a small effect size (*f*^2^ = 0.03).

**Fig. 2:**
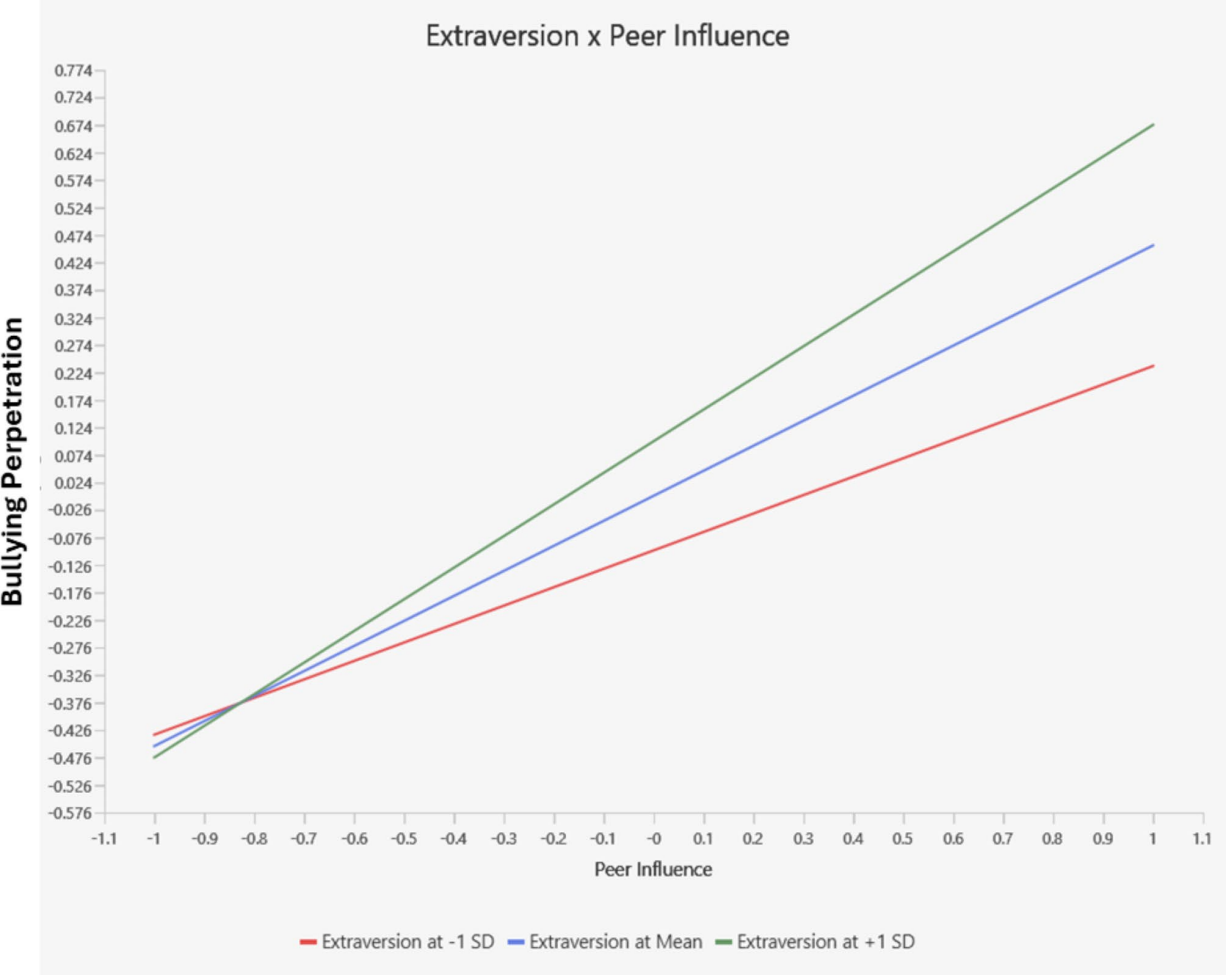
Interaction effect of peer influence and extraversion on bullying perpetration.

**Fig. 3:**
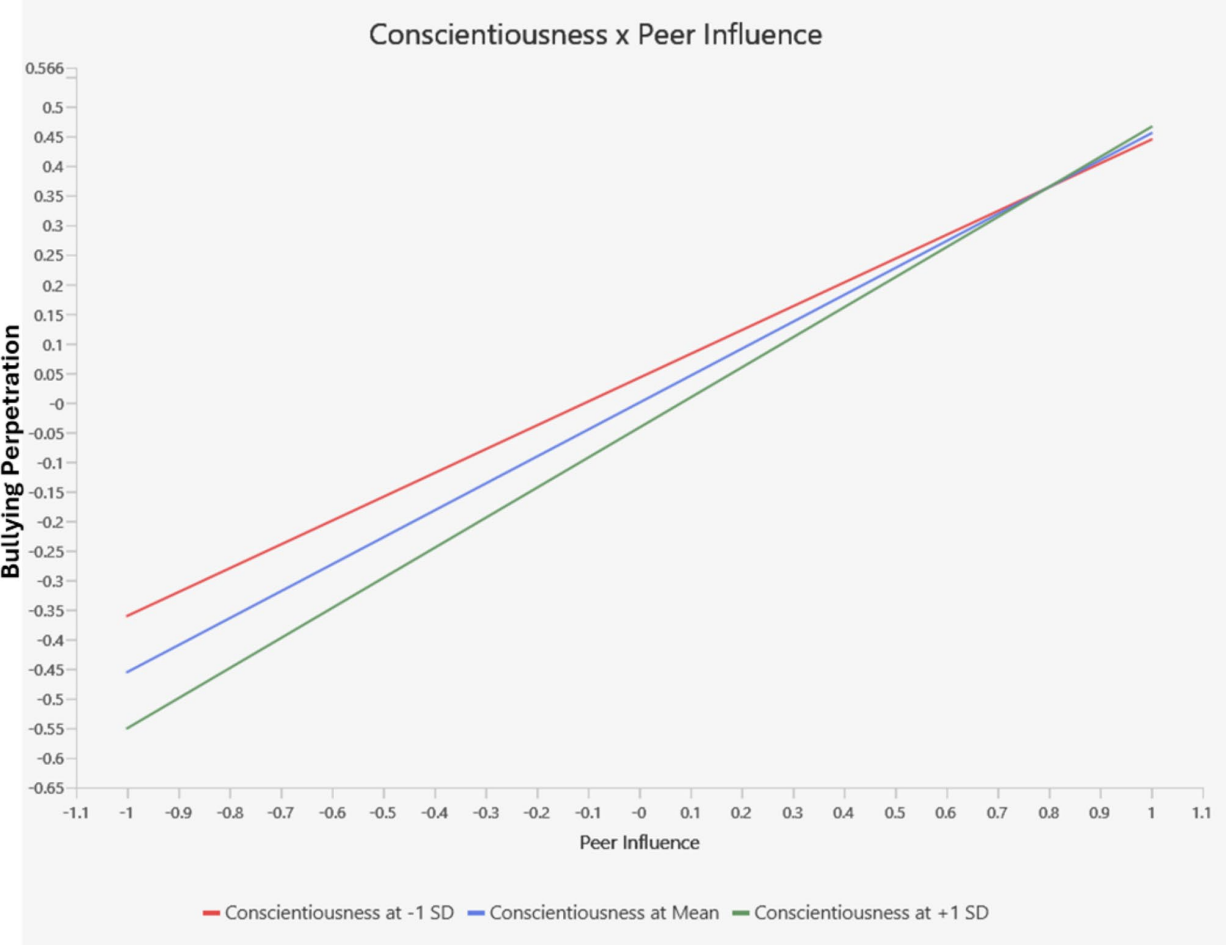
Interaction effect of peer influence and conscientiousness on bullying perpetration.

The moderating effects between Peer Influence and Personality Traits were examined to determine whether personality traits moderated the relationship between Peer Influence and Bullying Perpetration. The interaction between Extraversion and Peer Influence was significant (β = 0.12, *t* = 3.75, *p* < .001) (see Fig. 2), indicating that the effect of Peer Influence on Bullying Perpetration is stronger among adolescents with higher levels of Extraversion. Similarly, the interaction between Conscientiousness and Peer Influence was significant (β = 0.05, *t* = 2.03, *p* < .05), suggesting that the relationship between Peer Influence and Bullying Perpetration varies depending on the level of conscientiousness (see Fig. 3). The interactions involving Openness to Experience (β = -0.05, *t* = -1.55, *p* = .12), Emotional Stability (β = -0.03, *t* = -1.13, *p* = .26), and Agreeableness (β = 0.01, *t* = 0.36, *p* = .72) were not statistically significant, i ndicating that these personality traits did not significantly moderate the relationship between Peer Influence and Bullying Perpetration in this sample. The moderating effect of the personality traits indicates a very small effect size (*f*^2^ = 0.004).

### Mediation analysis

The mediating role of Peer Influence in the relationship between Childhood Adversity and Bullying Perpetration was tested using bootstrapping methods with 5,000 resamples. The indirect effects were significant for all forms of childhood adversity. For Punishment, the indirect effect through Peer Influence was significant (indirect effect = 0.05, 95% CI [0.03, 0.08], *p* < .001). Similarly, the indirect effects were significant for Sexual Abuse (indirect effect = 0.08, 95% CI [0.05, 0.11], *p* < .001) and Negative Home Atmosphere (indirect effect = 0.13, 95% CI [0.10, 0.16], *p* < .001). These results support Hypothesis 4, confirming that Peer Influence mediates the relationship between Childhood Adversity and Bullying penetration.

### Moderated mediation analysis

The moderated mediation analysis examined whether the indirect effect of Childhood Adversity on Bullying Perpetration through Peer Influence varied across levels of Personality Traits. The indices of moderated mediation were significant for Extraversion (Index of Moderated Mediation [IMM] = 0.12, *p* < .001) and Conscientiousness (IMM = 0.05, *p* < .05), indicating that these traits moderate the indirect effect.

At high levels of Conscientiousness (one standard deviation above the mean), the indirect effect of Negative Home Atmosphere on Bullying Perpetration through Peer Influence was significant and negative (β = -0.04, *t* = -2.90, *p* = .004). This suggests that high conscientiousness mitigates the effect of childhood adversity on bullying perpetration via reduced susceptibility to peer influence. At low levels of Conscientiousness (one standard deviation below the mean), the indirect effect was significant and positive (β = 0.06, *t* = 2.61, *p* = .009), indicating that low conscientiousness amplifies the adverse effect.

The conditional indirect effects involving Extraversion were not significant at either high or low levels, suggesting that Extraversion did not significantly moderate the indirect effect of Childhood Adversity on Bullying Perpetration through Peer Influence in this sample.

### Model fit for the moderated mediation model

The moderated mediation model demonstrated acceptable fit indices: χ²(480) = 810.25, *p* < .001; CFI = 0.95; RMSEA = 0.032; and SRMR = 0.045. These indices confirm that the model adequately fits the data.

The study’s findings highlight the significant roles of childhood adversity, peer influence, and personality traits in influencing bullying perpetration among South African adolescents. The results emphasise the importance of addressing adverse childhood experiences and susceptibility to peer pressure in interventions aimed at reducing bullying perpetration. Additionally, developing protective personality traits such as conscientiousness may help mitigate the impact of peer influence on bullying perpetration.

## Discussion

Bullying perpetration among adolescents is a complex issue influenced by a confluence of individual, social, and environmental factors. This study investigated the relationships between childhood adversity, peer influence, personality traits, and bullying perpetration among South African adolescents. The findings offer critical insights into the mechanisms underpinning bullying perpetration and have significant implications for interventions and future research.

The study confirmed that higher levels of childhood adversity—including punishment, sexual abuse, and a negative home atmosphere—are significantly associated with increased bullying perpetration (Hypothesis 1). This finding aligns with extensive literature indicating that adverse childhood experiences can lead to aggressive behaviours due to learned patterns and impaired emotional regulation^29,37^. In the South African context, where exposure to violence and abuse is relatively high^72^, these results underline the urgency of addressing childhood adversity to mitigate its long-term effects on adolescent behaviour.

From a theoretical perspective, Social Learning Theory^35^ suggests that children imitate behaviours observed in their environment. Adolescents exposed to violence or neglect may internalise these behaviours as acceptable means of interaction, leading to increased aggression and bullying. Furthermore, neurobiological studies have shown that early adversity can affect brain development, particularly in areas responsible for emotion regulation and impulse control^73^, which may predispose individuals to aggressive behaviours.

The findings also revealed that childhood adversity is positively associated with susceptibility to peer influence (Hypothesis 2). Adolescents who have experienced adversity may lack secure attachments and self-esteem, making them more likely to seek acceptance within peer groups and conform to group norms, including aggressive behaviours^74^. This susceptibility can be a coping mechanism to compensate for unmet emotional needs from adverse home environments.

Peer influence emerged as a significant predictor of bullying perpetration (Hypothesis 3). This is consistent with the idea that adolescents are highly influenced by their peers, especially during a developmental period when social acceptance is paramount^75^. In environments where aggression is normalised or rewarded, susceptible adolescents may engage in bullying to gain status or avoid rejection^44^. In South Africa, peer dynamics can be particularly influential due to collectivist cultural orientations in some communities, where group conformity is emphasised^76^.

The mediation analysis supported the hypothesis that peer influence mediates the relationship between childhood adversity and bullying perpetration (Hypothesis 4). This suggests that adverse childhood experiences increase vulnerability to negative peer influence, which in turn facilitates bullying behaviour. This mediated pathway highlights the necessity of addressing both individual histories of adversity and the social contexts in which adolescents operate.

Personality traits were found to moderate the relationships among childhood adversity, peer influence, and bullying perpetration (Hypothesis 5). Specifically, high conscientiousness and emotional stability were associated with lower levels of bullying, potentially serving as protective factors^77^. Adolescents with these traits may have better self-regulation and coping mechanisms, enabling them to resist negative peer pressures. Conversely, high extraversion was associated with increased bullying perpetration, possibly due to greater social engagement and a higher likelihood of being influenced by peers^78^.

The mediated moderation analysis (Hypothesis 6) revealed that the indirect effect of childhood adversity on bullying perpetration through peer influence varies depending on certain personality traits. Adolescents with high extraversion and low conscientiousness were more susceptible to peer influence leading to bullying, whereas those with high conscientiousness were less affected. This finding supports the Differential Susceptibility Hypothesis^79^, suggesting that individual differences influence how environmental factors impact behaviour.

### Practical implications

The findings have significant implications for developing targeted interventions to reduce bullying perpetration among South African adolescents.

Interventions should prioritise mitigating the effects of childhood adversity. Implementing trauma-informed care within schools can help identify and support students who have experienced adverse events. In the South African context, the “Journey of Hope” programme has been adapted to support children affected by HIV/AIDS and trauma, focusing on building resilience and coping strategies (Skeen et al., 2017). Such programmes could be expanded to address broader childhood adversities.

Moreover, community-based interventions like the “Sinovuyo Caring Families Programme” have effectively improved parenting practices and reduced child behaviour problems in South Africa^80^. By strengthening family relationships and reducing harsh parenting, these programmes can address the root causes of childhood adversity.

Given the significant role of peer influence, interventions promoting positive peer interactions are essential. The “WITS Programme” (*Walk Away*,* Ignore*,* Talk It Out*,* Seek Help*) has effectively reduced peer victimisation in Canada. It could be adapted for South African schools^81^. Additionally, the “Friendly Schools” initiative in Australia focuses on creating a positive school climate and enhancing peer support, which has reduced bullying behaviours^82^. Adapting such programmes to the South African context, with consideration for cultural complexities, could develop supportive peer environments.

Interventions aiming to develop protective personality traits can be beneficial. Social and Emotional Learning (SEL) programmes have successfully enhanced skills like self-awareness, self-management, social awareness, relationship skills, and responsible decision-making^83^. In South Africa, the “HealthWise South Africa” programme integrates SEL components to reduce risky behaviours among adolescents^84^. Expanding SEL programmes can help adolescents develop conscientiousness and emotional stability, reducing susceptibility to negative peer influence.

Considering the significant influence of the home environment, family-based interventions are crucial. The “Parenting for Lifelong Health” initiative provides free, evidence-based parenting programmes in low-resource settings, including South Africa, to reduce violence against children^72^. These programmes focus on positive parenting, improving parent-child communication, reducing harsh discipline, and addressing factors contributing to childhood adversity.

### Policy implications

At the policy level, enforcing laws against corporal punishment and child abuse is essential. Despite legal prohibitions, corporal punishment remains prevalent in South Africa^85^. Strengthening the implementation of existing laws and increasing public awareness about the harmful effects of corporal punishment can contribute to reducing childhood adversity. Additionally, policies promoting school-based mental health services can ensure that adolescents have access to support for emotional and behavioural issues.

### Limitations of the study

While the study provides valuable insights, certain limitations should be acknowledged. The cross-sectional design limits the ability to establish causality between variables. Longitudinal studies are needed to confirm the directional relationships and assess changes over time.

Additionally, the study focused on the Big Five personality traits. However, other personality dimensions, such as narcissism or callous-unemotional traits, which have been linked to aggressive behaviour^86^, were not examined. Exploring these traits could provide a more comprehensive understanding of individual differences in bullying perpetration.

Reliance on self-reported data may introduce bias due to social desirability or inaccurate recall. Incorporating multiple data sources, such as teacher reports, peer assessments, or behavioural observations, could enhance the validity of the findings.

### Recommendations for future research

Future research should explore the longitudinal effects of childhood adversity, peer influence, and personality traits on bullying perpetration. Longitudinal studies can provide insights into developmental trajectories and critical periods for intervention.

Investigating other personality traits beyond the Big Five, such as the Dark Triad traits (narcissism, Machiavellianism, psychopathy), which have been associated with bullying perpetration^87^, could deepen the understanding of individual factors contributing to bullying.

Additionally, it is essential to examine the effectiveness of culturally adapted intervention programmes within the South African context. For instance, research could evaluate the implementation of the “HealthWise South Africa” programme on bullying behaviours, as it incorporates components addressing peer influence and personal development^88^, and like KiVa, CBITS, and SEL curricula within South African schools, examining their impact on bullying behaviours and associated outcomes.

Exploring the impact of socio-economic factors and community violence on bullying perpetration can enhance understanding of environmental influences. Studies have shown that exposure to community violence is linked to increased aggression in adolescents^89^. Understanding these broader contextual factors can inform more comprehensive intervention strategies.

Furthermore, qualitative research involving in-depth interviews and focus groups with adolescents can provide broader insights into their experiences with bullying, peer relationships, and coping mechanisms. Such studies can inform the development of interventions that resonate with adolescents’ experiences and cultural contexts.

## Conclusion

This study highlights the complex relationship between childhood adversity, peer influence, and personality traits in predicting bullying perpetration among South African adolescents. By elucidating the mediating role of peer influence and the moderating effects of personality traits, the findings contribute to a holistic understanding of bullying perpetration. Interventions that holistically address these factors—mitigating childhood adversity, promoting positive peer interactions, and developing protective personality traits—are crucial for effectively reducing bullying and enhancing adolescent well-being in South Africa. Future research should continue to explore these dynamics and evaluate the effectiveness of culturally tailored interventions to inform policy and practice.

## Data Availability

Data for this study was deposited in the North-West University data repository (Dayta ya rona) (https://figshare.com/articles/dataset/Bullying_data_csv/25997566).
